# A meta-analysis of MMP-9 promoter −1562 C/T polymorphism on susceptibility of chronic periodontitis

**DOI:** 10.1186/s40064-016-2135-3

**Published:** 2016-04-26

**Authors:** Deepal Haresh Ajmera, Pradeep Singh, Ying Zhu, Wenyang Li, Jinlin Song

**Affiliations:** College of Stomatology, Chongqing Medical University, Chongqing, 400016 China; Chongqing Key Laboratory of Oral Diseases and Biomedical Sciences, Chongqing, China; Chongqing Municipal Key Laboratory of Oral Biomedical Engineering of Higher Education, Chongqing, China; Department of Forensic Medicine, Faculty of Basic Medical Sciences, Chongqing Medical University, Chongqing, China; Department of Oral and Maxillofacial Surgery, College of Stomatology, Chongqing Medical University, Chongqing, China

**Keywords:** Matrix metalloproteinase-9, Polymorphism, Chronic periodontitis, Meta-analysis

## Abstract

**Background:**

Although many studies have focused on the association of the *MMP*-9 promoter −1562 C/T polymorphism with the susceptibility and/or severity of chronic periodontitis (CP), results have been inconsistent. Therefore, a meta-analysis of all eligible studies was performed to derive a more precise estimation of the association between this polymorphism and CP risk.

**Methods:**

All relevant studies were identified through a database search in PubMed, Medline, and Web of Science. All the full-text studies with appropriate analytical design, published in English, which evaluated the association of *MMP*-*9* promoter −1562C/T polymorphism with CP risk using validated genotyping methods, and with non-duplicated data were selected for this study. A fixed-effect model was used to calculate pooled ORs in the absence of heterogeneity across included trials (P > 0.1 and I^2^ < 50 %), otherwise the random-effect model was applied.

**Results:**

In an overall meta-analysis, pooled ORs revealed that T variant in the *MMP*-*9* promoter −1562 C/T polymorphism was associated with a significantly decreased risk for CP under all comparison models. In subgroup analyses by ethnicity, pooled ORs showed that a significant association of the *MMP*-*9* promoter −1562 C/T polymorphism with CP risk was only detected in Caucasians and Asians but not in mixed population. In the subgroup analysis by severity of CP, pooled ORs indicated that T allele of the *MMP*-*9* promoter −1562 C/T polymorphism was associated with decreased susceptibility to severe CP while there was no significant association between this polymorphism and moderate CP.

**Conclusions:**

Our meta-analysis showed that T allele in the *MMP*-*9* promoter −1562 C/T polymorphism might be a protective factor for CP, especially in Caucasians and Asians. Moreover, there was a significant association of the MMP-9 promoter −1562 C/T polymorphism with decreased susceptibility to severe CP, while the allelic and/or genotype distributions of this polymorphism were not associated with moderate CP.

**Electronic supplementary material:**

The online version of this article (doi:10.1186/s40064-016-2135-3) contains supplementary material, which is available to authorized users.

## Background

Chronic periodontitis (CP), the most commonly occurring and slowly progressive form of periodontal disease, can lead to continual inflammatory host response, which may finally result in periodontal attachment loss and bone resorption (Deo and Bhongade 2010; Pihlstrom et al. 2005). CP is a highly prevalent disease and has shown to affect 90 % of the worldwide individuals (Tatakis and Kumar 2005). Even though the key etiological factor that results in progression of CP is the formation of complex biofilm on the surfaces of teeth adjacent to their periodontal tissues, determinants like demographic, social, environmental, behavioral, systemic and genetic factors have also been coupled with the epidemiology of this disease (Stabholz et al. 2010; Yoshie et al. 2007).

Matrix metalloproteinases (MMPs) belong to a family of zinc-dependent endopeptidases, which are served as the vital enzymes. They have the combined capacity to degrade extracellular matrix (ECM) components such as several kinds of collagen, that are essential for the tissue repair and remodeling involved with the development of inflammation (Hannas et al. 2007; Sorsa et al. 2006). MMPs are secreted in an inactive form as pro-MMPs, which can be activated by other MMPs or serine proteinases (Malemud 2006; Sternlicht and Werb 2001). The imbalance between MMPs, identified in the inflamed periodontal tissues and their host inhibitors is thought to be responsible for the process of destruction in structural proteins during CP (Emingil et al. 2006; Ingman et al. 1996; Victor et al. 2014).

Matrix metalloproteinase-9 (MMP-9) or gelatinase B, involved in the breakdown of various connective tissue proteins, including types IV, V and XI collagen, proteoglycans as well as elastin, is abundantly expressed in CP (Bildt et al. 2008; Rai et al. 2008). Various cell lines such as polymorphonuclear leukocytes, macrophages, keratinocytes, fibroblasts, osteoclasts, eosinophils and neutrophils have been connected with the expression of *MMP*-*9* gene, located on chromosome 20q11.2-13.1 (Seguier et al. 2001). Genetic variations in the promoter region of *MMP*-*9* gene may have an effect on its transcription and protein synthesis (Chang et al. 2002; Soder et al. 2009), which may influence connective tissue degradation of the protein and thus contributing to genetic susceptibility to CP.

The fact that a functional C-to-T single nucleotide polymorphism (SNP) exists in MMP-9 gene at position −1562, which affects transcription, and also decreased transcriptional activity shown by CC genotype is well documented (Haberbosch and Gardemann 2005; Zhang et al. 1999). Despite comprehensive studies focusing on the association of this polymorphisms with the susceptibility and/or severity of CP using the similar methodology(de Souza et al. 2005; Gurkan et al. 2008; Holla et al. 2006; Keles et al. 2006), the results show high degree of variation. Therefore a meta-analysis of all eligible studies was carried out to draw an accurate assessment of the association of this polymorphism with CP risk.

## Methods

### Protocols and eligibility criteria

The meta-analysis and systematic review reported here is in accordance with the PRISMA—Preferred Reporting Items for Systematic Review and Meta-analyses (Additional file 1: Appendix S1). The formulated research question follows the Population, Intervention, Comparison, Outcomes (PICO) criteria. The literature search included all the potential human studies on the association of Matrix metalloproteinases SNPs with periodontitis risk.

### Search strategy

All relevant studies were identified through a search in the databases (updated to 6 April 2015) of PubMed, Medline, and Web of Science, with combinations of the following terms used as MESH headings and free text words: (“matrix metalloproteinase-9” or “gelatinase B” or “MMP-9” or “MMP9”) and (“genetic variant” or “genetic variation” or “polymorphism”) and (“periodontitis” or “chronic periodontitis” or “CP” or “periodontal disease” or “PD”). We limited all searches to clinical trial, meta-analysis as well as review. In addition, any potentially relevant papers that may have been missed during the process of computer-assisted searches were also identified via the manual search of bibliography lists.

### Selection of studies

The following criteria were designed and used for including the identified studies into the present meta-analysis and system review: (1) Studies that evaluated the association of *MMP*-*9* promoter −1562 C/T polymorphism with CP risk among CP affected and unaffected individuals; (2) Studies applied validated genotyping methods such as PCR–RFLP; (3) Studies with appropriate analytical design, for example case–control, cohort, or nested case control; (4) Studies published in English, and available full-text; (5) Study data not duplicated or overlapped with those of any other article. Besides, we barred those studies, of which the pertinent data were not available to figure up the odds ratios (ORs) and its variance.

### Data extraction

The data extraction was performed by two independent reviewers (ADH and PS) under a pre-defined strategy. Any disagreements between investigators were settled through consensus decision with the third evaluator (LWY). The following items were collected from each included trial: first author’s surname, publication year, nation, race, sample size, severity of CP, matching criteria, genotyping method, as well as the results of HWE in controls were calculated using an online software (http://ihg2.helmholtz-muenchen.de/cgi-bin/hw/hwa1.pl).

### Heterogeneity

Heterogeneity across the included studies was evaluated by the Cochrane-Q test. *I*^2^ directly calculated from the Cochrane-Q test was also used to describe the presence of inconsistency among the included studies caused by heterogeneity. The range of *I*^2^ varies from 0 to 100 %, with 0 % suggesting that there is no heterogeneity. Moreover, low heterogeneity was assumed if *I*^2^ was below 50 %, while, if *I*^2^ exceeded 50 %, a significant heterogeneity was believed to exist.

### Statistical analysis

The association of the *MMP*-*9* promoter −1562 C/T polymorphism with CP risk was assessed by ORs with their 95 % confidence intervals (CIs). The Z-test was applied to find out statistical significance of pooled ORs. Initially, the allele-frequency comparison model (T vs. C) was used to evaluate the potential relationship between the allele T of *MMP*-*9* promoter −1562 C/T polymorphism and CP risk. After that, the association of this polymorphism with CP risk was also examined by four other comparison models, including TT versus CC, CT versus CC, dominant (TT + CT vs. CC), as well as recessive (TT vs. CT + CC) genetic models. In addition, subgroup analyses concerning the study characteristics of ethnicity and severity of CP were respectively carried out.

To calculate pooled ORs, a fixed-effects model was planned to be used with the absence of heterogeneity across included trials (P > 0.1 and *I*^2^ < 50 %), otherwise, the random-effects model was applied. Furthermore, the potential source of heterogeneity was evaluated via a sensitivity analysis. Besides, we used funnel plots to examine publication bias, in which a standard error of log (OR) of each included trial was plotted against its log (OR), and Egger’s linear regression test was applied to assess the asymmetry. Meta-analyses was executed by using software Stata (version 11.0, Stata Corp., College Station, TX), with a statistical significance defined as two-sided P value <0.05. If the results of trials could not be pooled through the meta-analysis, we assessed them using descriptive statistics.

## Results

**Fig. 1:**
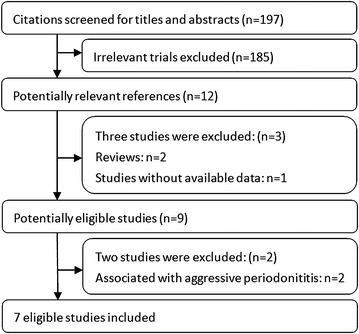
Flow of study identification, inclusion, exclusion

The workflow and results of literature search are summarized in Fig. 1. The comprehensive search of literature under defined terms retrieved 197 articles. Of those 185 articles were barred due to the irrelevance to this topic, after a thorough screening of titles and abstracts. After the detailed screening of 12 potentially relevant references, 9 articles were taken for a further full-text review. Following full-text retrieval of the articles, two studies were eliminated as they investigated the association of the *MMP*-*9* promoter −1562 C/T polymorphism with aggressive periodontitis (Chen et al. 2007; Gurkan et al. 2007). Finally, seven case–control studies involving a total of 859 CP cases and 1186 controls were included in this meta-analysis (de Souza et al. 2005; Gurkan et al. 2008; Holla et al. 2006; Isaza-Guzman et al. 2011; Keles et al. 2006; Li et al. 2012; Loo et al. 2011). The baseline characteristics of all the included studies are summarized in Table 1. As far as stratification by ethnicity is concerned, 3 studies reported on Caucasians, 2 on Asians, and 2 on subjects with mixed populations were included in the ultimate analysis. Also, concerning the severity of CP, the final analysis included two studies (1 reported on Caucasians and 1 on mixed populations) that investigated the association of *MMP*-*9* promoter −1562 C/T polymorphism with risk for both moderate CP and severe CP. Moreover, the genotype distributions of all the included studies were in accordance with HWE except for the study of Gurkan et al. (2008), Loo et al. (2011) and Li et al. (2012).

**Table 1:** Baseline characteristics of studies included for investigating the association of the *MMP*-*9* promoter −1562 C/T polymorphism with periodontitis risk

References	Country	Ethnicity	Sample size (case/control)	Type of periodontitis	Matching criteria	Genotype method	HWE in controls
de Souza et al. (2005)	Brazil	Mixed	62/38	Moderate or severe CP	–	PCR–RFLP	0.623
Holla et al. (2006)	Czech	Caucasian	169/135	Moderate or severe CP	Age, gender, smoker ratios	PCR–RFLP	0.586
Keles et al. (2006)	Turkey	Caucasian	70/70	Severe CP	Age, gender	PCR–RFLP	0.816
Gurkan et al. (2008)	Turkey	Caucasian	87/107	Severe CP	Age, gender,smoker ratios	PCR–RFLP	0.017
Loo et al. (2011)	China	Asian	280/250	Severe CP	Age, gender	PCR–RFLP	0.001
Isaza-Guzman et al. (2011)	Colombia	Mixed	69/54	Slight to moderate to severe CP	Gender	PCR–RFLP	0.163
Li et al. (2012)	China	Asian	122/532	Severe CP	–	PCR–RFLP	0.001

*MMP*-*9* matrix metalloproteinase-9, CP chronic periodontitis, *GAgP* generalized aggressive periodontitis, *HWE* Hardy–Weinberg equilibrium

Slight periodontitis: patients with teeth exhibiting <3 mm attachment loss; moderate periodontitis: patients with teeth exhibiting ≥3 and <7 mm attachment loss; severe periodontitis: patients with teeth exhibiting ≥7 mm attachment loss

A P value less than 0.05 of HWE was considered significant

### Meta-analysis results

**Fig. 2:**
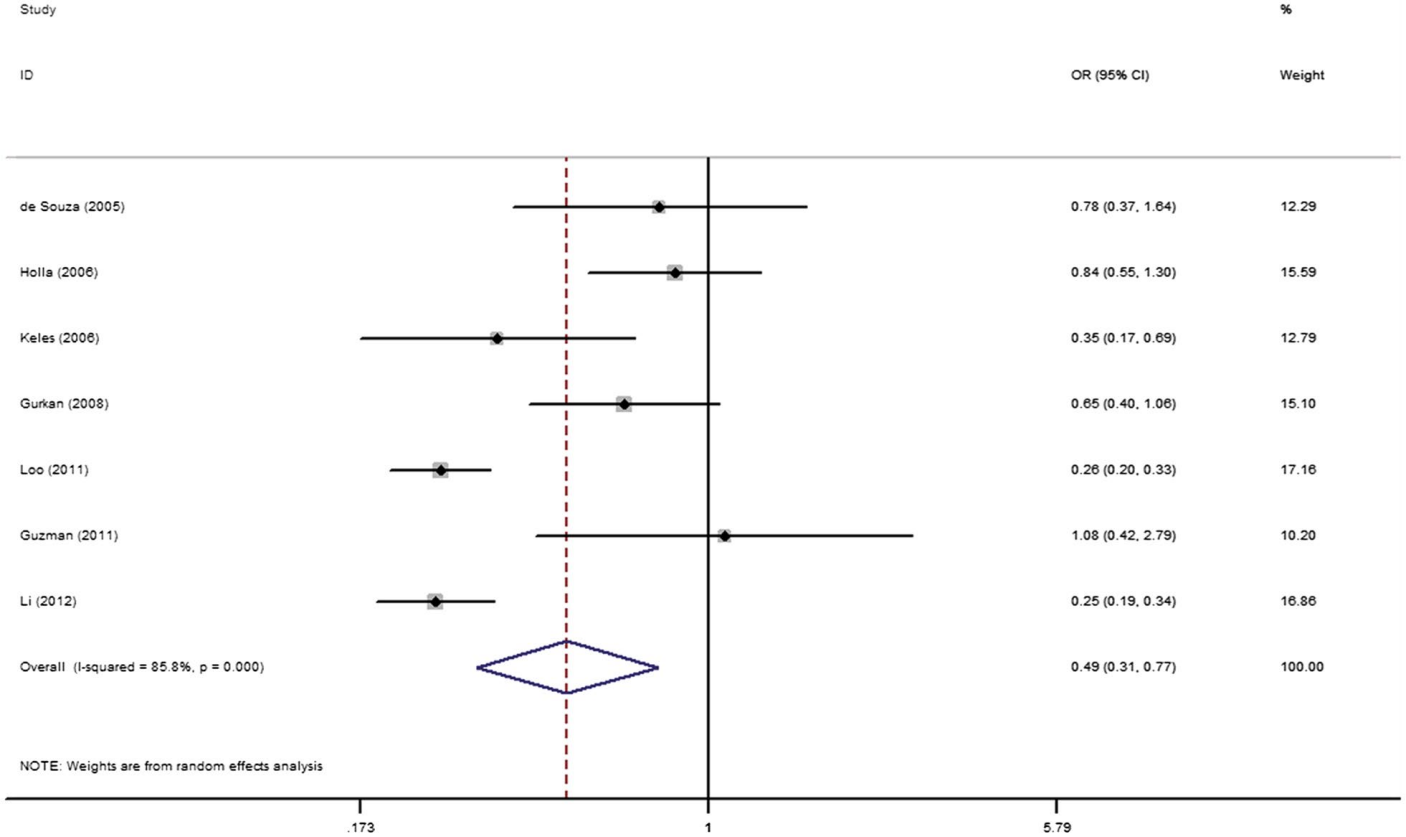
Forest plot of CP risk associated with the *MMP*-*9* promoter −1562 C/T polymorphism under the allele contrast

As shown in Table 2 and Fig. 2, the allele-frequency comparison model produced a significant relation between the *MMP*-*9* promoter −1562 C/T polymorphism and CP risk (T vs. C: OR 0.49, 95 % CI 0.31–0.77, P = 0.002), but with a considerable heterogeneity among the included studies (P_h_ = 0.000, *I*^2^ = 85.8 %) (Table 2; Fig. 2). Besides, statistically significant relation of this polymorphism with CP risk was also identified under all other different genetic models (Table 2).

**Table 2:** Meta-analysis results of the association of the *MMP*-*9* promoter −1562 C/T polymorphism with CP risk

*MMP*-*9* (−1562 C/T)	Studies (cases/controls)	T versus C OR (95 % CI), P P_h_, *I* ^2^ (%)	TT versus CC OR (95 % CI), P P_h_, *I* ^2^ (%)	CT versus CC OR (95 % CI), P P_h_, *I* ^2^ (%)	Dominant genetic model OR (95 % CI), P P_h_, *I* ^2^ (%)	Recessive genetic model OR (95 % CI), P P_h_, *I* ^2^(%)
Total	7 (859/1186)	0.49 (0.31–0.77), 0.002 0.001, 85.8	0.16 (0.12–0.22), 0.001 0.564, 0.0	0.53 (0.33–0.85), 0.008 0.001, 73.4	0.46 (0.26–0.82), 0.009 0.001, 85.8	0.27 (0.20–0.35), 0.001 0.897, 0.0
Ethnicity						
Caucasian	3 (326/312)	0.61 (0.39–0.97), 0.037 0.101, 56.3	0.35 (0.13–0.97), 0.044 0.452, 0.0	0.66 (0.47–0.92), 0.016 0.227, 32.5	0.62 (0.45–0.87), 0.005 0.139, 49.3	0.40 (0.14–1.10), 0.076 0.535, 0.0
Asian	2 (402/782)	0.26 (0.21–0.31), 0.001 0.902, 0.0	0.14 (0.10–0.20), 0.001 0.926, 0.0	0.27 (0.19–0.39), 0.001 0.522, 0.0	0.19 (0.14–0.26), 0.001 0.756, 0.0	0.26 (0.20–0.35), 0.001 0.782, 0.0
Mixed	2 (131/92)	0.89 (0.49–1.59), 0.684 0.596, 0.0	0.23 (0.02–2.25), 0.206 0.883, 0.0	1.09 (0.56–2.11), 0.808 0.453, 0.0	0.98 (0.51–1.88), 0.953 0.512, 0.0	0.23 (0.02–2.22), 0.202 0.915, 0.0
Severity of chronic periodontitis						
Severe	6 (687/1132)	0.46 (0.28–0.75), 0.002 0.001, 87.6	0.16 (0.12–0.22), 0.001 0.260. 23.2	0.48 (0.29–0.81), 0.006 0.001, 75.9	0.41 (0.21–0.78), 0.006 0.001, 87.2	0.27 (0.21–0.36), 0.001 0.680, 0.0
Moderate	2 (103/173)	0.65 (0.40–1.07), 0.090 0.358, 0.0	0.34 (0.06–2.08), 0.245 0.831, 0.0	0.70 (0.39–1.24), 0.218 0.319, 0.0	0.65 (0.37–1.14), 0.132 0.323, 0.0	0.37 (0.06–2.26), 0.284 0.891, 0.0

*MMP*-*9*: *matrix metalloproteinase*-*9*; CP: chronic periodontitis; dominant genetic model: TT + CT versus CC; recessive genetic model: TT versus CT + CC; P_h_: the P value of heterogeneity; OR: odds ratio; CI: confidence interval

When P_h_ is < 0.1 and *I* ^2^ exceeded 50 %, the random-effects modelwas used. Conversely, the fixed-effects model was used

### Subgroup analysis

**Fig. 3:**
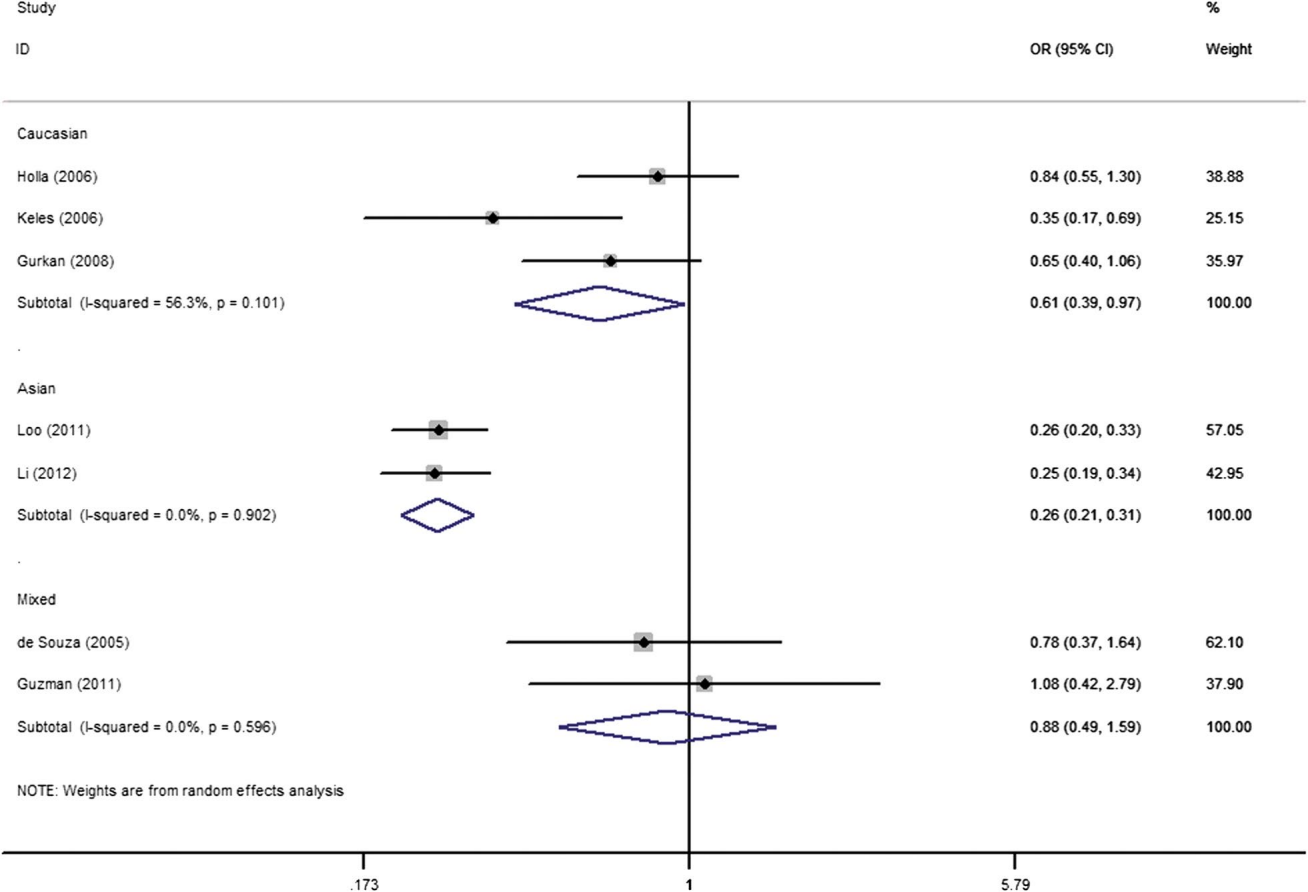
Forest plots demonstrating the association of *MMP*-*9* promoter −1562 C/T polymorphism to CP risk in regards to the ethnicity under the allele contrast

**Fig. 4:**
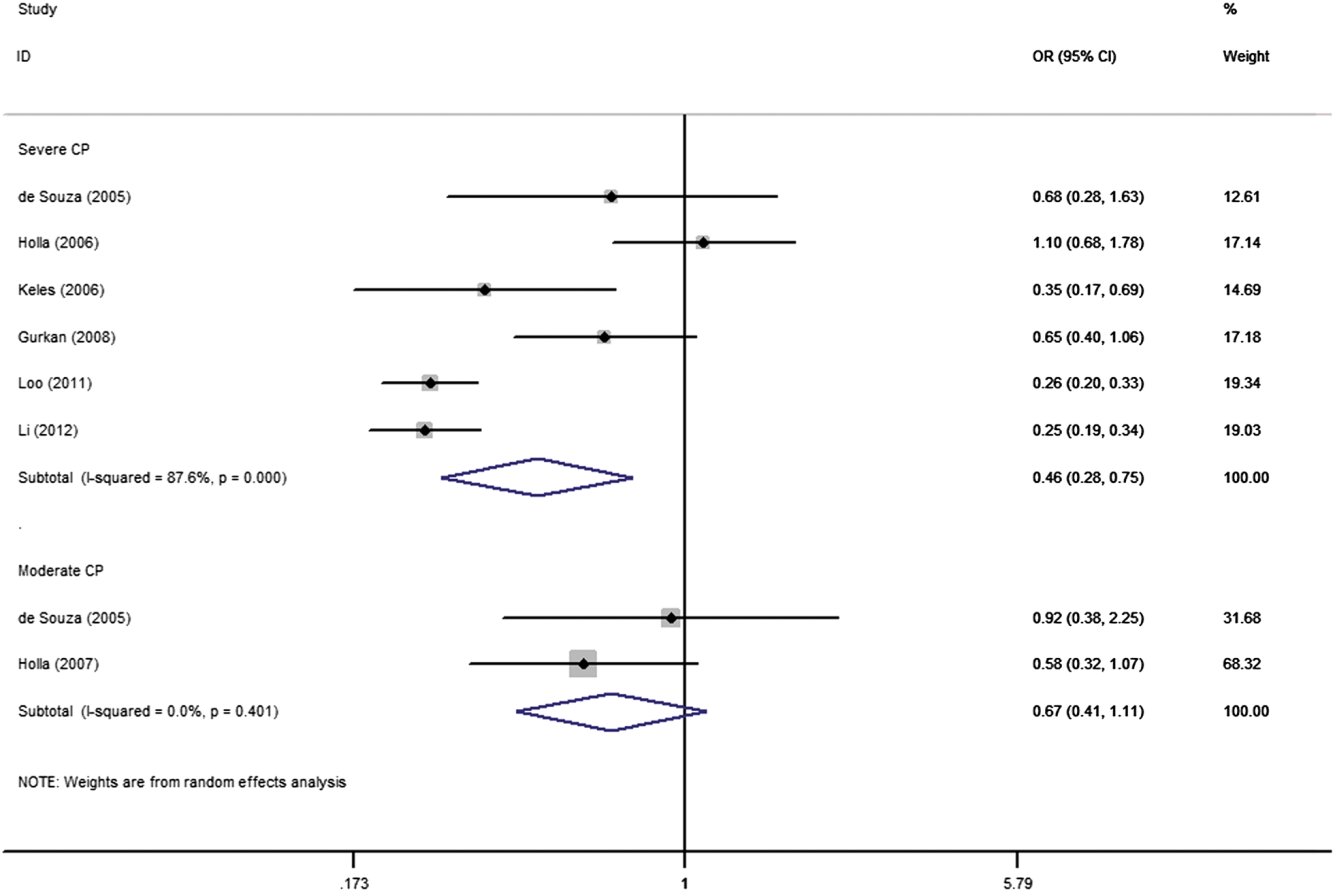
Forest plot illustrating the association between *MMP*-*9* promoter −1562 C/T polymorphism and CP risk with regards to the severity of CP under the allele contrast

We performed subgroup analysis according to the ethnicity and severity of CP. We found that the *MMP*-*9* promoter −1562 C/T polymorphism was associated with a significantly decreased CP risk under all comparison models except for the recessive genetic model in Caucasians (Table 2; Fig. 3). Besides, pooled analysis using the fixed-effects model for subgroup analysis by ethnicity revealed a significant association between this polymorphism and CP susceptibility in Asians under all comparison models (Table 2; Fig. 3). However, under all comparison models, we failed to detect any significant association between this polymorphism and CP risk in a mixed population (Table 2; Fig. 3). As long as the severity of CP was concerned, significant differences were detected in the allelic and/or genotype distributions of the *MMP*-*9* promoter −1562 C/T polymorphism between a subgroup of patients with severe CP versus controls (Table 2; Fig. 4), while under all comparison models the overall OR with its 95 % CI demonstrated that the difference between this polymorphism in patients with moderate CP and control population was not significant (Table 2; Fig. 4).

### Sensitivity analysis

Sensitivity analyses indicated that the major contributor of heterogeneity in different comparison models was the study by Li et al. (2012). After exclusion of this study, pooled ORs of the remaining trials revealed similar results except under the recessive genetic model (OR 0.54, 95 % CI 0.29–1.01, P = 0.054) (Table 3). Moreover, when we performed population-specific and severity-specific sensitivity analyses, we found that studies conducted by Keles et al. (2006) and Holla et al. (2006) were the main contributor of heterogeneity in subgroups of Caucasians and severity CP, respectively. The exclusion of the study by Keles et al. (2006) could eliminate the heterogeneity, and using a fixed-effects model, pooled ORs showed that there was no significant association between the *MMP*-*9* promoter −1562 C/T polymorphism and CP risk in Caucasian population under the allele-frequency comparison model (OR 0.75, 95 % CI 0.54–1.04, P = 0.080) (Table 3). Besides, with the exclusion of study by Holla et al. (2006), pooled ORs of the remaining trials in the subgroup of severe CP still showed similar results (Table 3).

**Table 3:** Sensitivity analysis for heterogeneity

MMP-9 (−1562 C/T)	Study majorly contributed to the heterogeneity	Heterogeneity after excluding the study		Pooled ORs of the remaining studies
		P_h_	*I* ^2^, %	OR 95 % CI, P
Total				
T versus C	Li et al.’s study	0.000	85.2	0.56 (0.33, 0.97), 0.040
CT versus CC	Li et al.’s study	0.013	65.4	0.62 (0.39, 0.98), 0.040
Dominant genetic model	Li et al.’s study	0.000	82.9	0.54 (0.29, 1.01), 0.054
Ethnicity				
Caucasian				
T versus C	Keles et al.’s study	0.440	0.0	0.75 (0.54, 1.04), 0.080
Severity of chronic periodontitis				
Severe				
T versus C	Holla et al.’s study	0.003	75.2	0.37 (0.25, 0.54), 0.000
CT versus CC	Holla et al.’s study	0.108	47.3	0.36 (0.28, 0.47), 0.000
Dominant genetic mode	Holla et al.’s study	0.003	75.2	0.32 (0.19, 0.53), 0.000

MMP-9: matrix metalloproteinase-9; dominant genetic model: TT + CT versus CC; P_h_: the P value of heterogeneity; OR: odds ratio; CI: confidence interval

### Publication bias

**Fig. 5:**
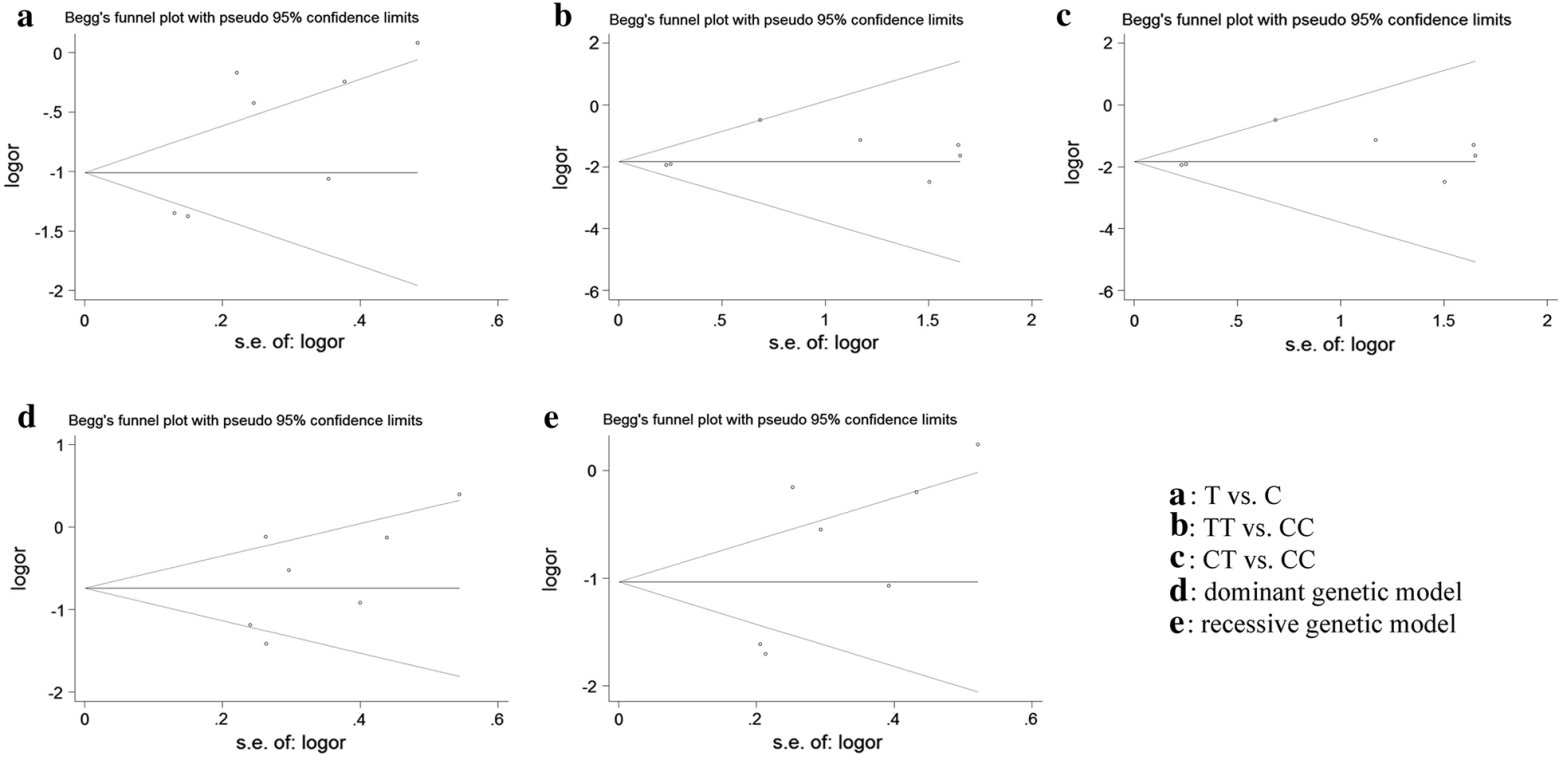
Begg’s funnel plot of the *MMP*-*9* promoter −1562 C/T polymorphism and CP risk in different contrast models. A: T versus C, B: TT versus CC, C: CT versus CC, D: dominant genetic model (TT + CT vs. CC), and E: recessive genetic model (TT vs. CT + CC)

In order to assess the publication bias of the included trials, Begg’s funnel plot and Egger’s test were performed. Funnel plot shapes were found to be symmetrical in all the comparison models of the *MMP*-*9* promoter −1562 C/T polymorphism (Fig. 5). In addition, for the purpose of providing statistical evidence to funnel plot symmetry, Egger’s test was used in this study. In the end, the results of the tests once again suggested that no publication bias existed for the association of this polymorphism (P = 0.057 for allele-frequency model, P = 0.295 for TT versus CC, P = 0.295 for CT versus CC, P = 0.213 for the dominant genetic model, P = 0.103 for the recessive genetic model) with CP risk.

## Discussion

Current understanding of the etiology of CP indicates the bacterial infection by a group of predominantly gram-negative and anaerobic organisms as the essential cause for disease initiation (Flemmig 1999), however, different evolution and the poor prognosis of severe CP cannot be justified by mere existence, type or quantity of infection. And the severity of periodontal destruction is rather dependent on a dynamic equilibrium of bacteria–host interactions that can be modified by multiple environmental and genetic factors (Page et al. 1997). Therefore, the effect of host genetic factors on disease susceptibility, progression and treatment outcome of CP has been suggested by many authors (Michalowicz et al. 2000; Offenbacher 1996). Evidence from these studies has implicated that about half of the variations found in the expression of CP can be attributed to genetic factors (Michalowicz et al. 2000). As a result, the need for searching some specific genetic markers for CP is on the rise.

A single nucleotide polymorphism (rs3918242) in the promoter region of *MMP*-*9* gene has been reported at position −1562 relative to the transcription start site, where a conversion between C and T (C−1562-T) occurs (Zhang et al. 1999). This functional polymorphism causes loss of binding of a nuclear repressor protein, thereby resulting in an increase of mRNA, protein level and MMP-9 activity in T allele carriers while the CC genotype has been shown to decrease transcriptional activity (Zhang et al. 1999).

Moreover, it has been proved that inappropriate activation of MMP-9 at a site of infection can have a profound effect in the tissue destruction of CP (Rai et al. 2008; Soder et al. 2009). However, studies about the association between this polymorphism and CP risk have not yielded consistent results. Several studies have supported that risk for CP is associated with this polymorphism (Keles et al. 2006; Li et al. 2012; Loo et al. 2011), whereas others have failed to find an association (de Souza et al. 2005; Gurkan et al. 2008; Holla et al. 2006; Isaza-Guzman et al. 2011).

Therefore, we performed the present meta-analysis to clarify the relationship between this polymorphism and CP risk. In this study, we first summarized the data about the association between the *MMP*-*9* promoter −1562 C/T polymorphism and CP risk in overall population. Surprisingly, pooled ORs revealed that T variant in the *MMP*-*9* promoter −1562 C/T polymorphism was associated with a significantly decreased risk for CP under all comparison models. The possible explanation might be that the MMP-9 expression is primarily controlled at the transcriptional level, where the promoter of *MMP*-*9* gene responds to stimuli of various cytokines and growth factors (Kondapaka et al. 1997). Furthermore, the T allele of this variant can abolish a binding site for a transcription repressor and thus change the promoter activity of *MMP*-*9*, leading to increased MMP-9 expression. Besides, an exchange of C-to-T at position −1562 can also alter the binding of a nuclear protein to this region, resulting in increased transcriptional activity in macrophages (Zhang et al. 1999). Therefore, it can be suggested that carriage of T allele may down-regulate the transcription activity and thus decreases the expression level of MMP-9 protein, which may offer a lower susceptibility of CP. In conclusion, *MMP*-*9* promoter −1562 C/T polymorphism may be a good biomarker for diagnosis and prognosis of CP.

In the subgroup analysis by ethnicity, pooled ORs revealed that a significant association of the *MMP*-*9* promoter −1562 C/T polymorphism with CP risk was only detected in Caucasian and Asian populations but not in mixed population. The reasonable explanation for this would be, firstly, the mixed population included not only Caucasians and Asians but also a large ethnic mixture of mestizos (white-Amerindian mix), Latin-European whites, mulattoes (black-white mix), zambos (black-Amerindian mix), blacks (Afro-Americans), and pure indigenous Amerindians (de Souza et al. 2005; Isaza-Guzman et al. 2011). Therefore, the distribution of alleles and/or genotypes is often different in different races. Secondly, distinct races having different risk alleles for a different phenotype which can exhibit different genotypic frequencies which may be influenced by sampling methods, racial and regional factors that may complicate interpretation of the results of genetic studies (Gurkan et al. 2008; Loos et al. 2005). It can also be speculated that there might be other transcription and/or activity, such as other regulatory elements, or promoter methylation (Cotignola et al. 2007) responsible for these contrasting results. Also, functionality of this polymorphism may depend on interaction with other factors such as genetics, environmental, tissue remodeling, and bacterial pathogens, to modulate susceptibility to disease causing increased MMP-9 expression (Demacq et al. 2008; Holla et al. 2006). Therefore, possible positive associations between a genetic marker and disease within one population may not necessarily be extrapolated to other populations. Based on these results, we can make a conclusion that the etiology of CP is genetically heterogenous.

In the subgroup analysis by severity of CP, the results indicated that T allele of the *MMP*-*9* promoter −1562 C/T polymorphism was associated with decreased susceptibility to severe CP while there was no significant association between this polymorphism and moderate CP. Our results were in accordance with the previous studies (Gurkan et al. 2008; Keles et al. 2006); however, more convincing evidence, such as larger sample size and number of studies, is still required to draw a more substantial conclusion. In combination with our results of the overall analysis, this subgroup analysis may lead to a conclusion that the *MMP*-*9* promoter −1562 C/T polymorphism not only contributes to the disease susceptibility but also the severity of CP.

Most of the evidence from our study should be considered to be stable and convincing albeit substantial heterogeneity since the heterogeneity detected in many pooled analyses did not have a significant impact on the results of our study. However, some potential limitations still exist in our study that needs to be addressed before interpreting these results. Firstly, a relatively large heterogeneity was evident in this meta-analysis. Nevertheless, through stratified analysis by ethnicity and severity of CP, heterogeneity reduced significantly. As a result, we presumed that relatively large heterogeneity mainly results from differences in ethnicity and severity of CP. Secondly, our overall outcomes were based on individual unadjusted ORs. A more precise analysis would have been conducted by adjustment of other covariates, such as age, gender, smoking status, environmental factors, and etc. thereby avoiding serious confounding bias. Accordingly, control group individuals might develop CP later within the age range, if unmatched by age and gender, as seen in some studies(de Souza et al. 2005; Li et al. 2012) hence underestimating the OR association with the genotype. Moreover, further evaluation of potential interactions between gene-to-environment, different polymorphic loci of the same gene, and even gene-to-gene, was limited due to unavailability of original data of the included studies, all of which may modulate CP risk. Finally, in our meta-analysis, a relatively small number of studies and sample size was used. Also, our meta-analysis included only those studies which were published in English. Therefore, it is possible that some relevant studies published in other languages were not included, which might introduce publication bias. The results for publication bias were statistically insignificant in our study though.

In conclusion, our meta-analysis showed that T allele in the *MMP*-*9* promoter −1562 C/T polymorphism might be a protective factor for CP, especially in Caucasian and Asian populations. Moreover, there was a significant association of the *MMP*-*9* promoter −1562 C/T polymorphism with decreased susceptibility to severe CP, while there was no association seen between the allelic and/or genotype distributions of this polymorphism and moderate CP. However, more studies of high quality with larger sample sizes and also other ethnic populations are still needed to draw a more solid inference on the relation between this polymorphism and CP risk.

## Additional file

10.1186/s40064-016-2135-3 The Preferred Reporting Items for Systematic Review and Meta-Analyse (PRISMA) statement of our study.
